# Reversible Data Hiding Algorithm in Fully Homomorphic Encrypted Domain

**DOI:** 10.3390/e21070625

**Published:** 2019-06-26

**Authors:** Jingxuan Li, Xingyuan Liang, Ceyu Dai, Shijun Xiang

**Affiliations:** College of Information Science and Technology, Jinan University, Guangzhou 510632, China

**Keywords:** reversible data hiding, DGHV, public key cryptosystem, information entropy, cloud computing

## Abstract

This paper proposes a reversible data hiding scheme by exploiting the DGHV fully homomorphic encryption, and analyzes the feasibility of the scheme for data hiding from the perspective of information entropy. In the proposed algorithm, additional data can be embedded directly into a DGHV fully homomorphic encrypted image without any preprocessing. On the sending side, by using two encrypted pixels as a group, a data hider can get the difference of two pixels in a group. Additional data can be embedded into the encrypted image by shifting the histogram of the differences with the fully homomorphic property. On the receiver side, a legal user can extract the additional data by getting the difference histogram, and the original image can be restored by using modular arithmetic. Besides, the additional data can be extracted after decryption while the original image can be restored. Compared with the previous two typical algorithms, the proposed scheme can effectively avoid preprocessing operations before encryption and can successfully embed and extract additional data in the encrypted domain. The extensive testing results on the standard images have certified the effectiveness of the proposed scheme.

## 1. Introduction

Reversible data hiding [1,2,3] is an efficient technology that combines the robustness and provability of digital information, and embeds information for authentication. The hidden data can be extracted completely and the original carrier can be restored completely after data extraction. Because of the existence of these characteristics, reversible data hiding has been applied in many areas such as business and military. The most basic reversible data hiding technology dealt with the redundancy of digital information and then embedded the additional data. There are several reversible data hiding algorithms using difference expansion [4,5,6], histogram shifting [7,8,9,10] and the new prediction error algorithms have higher payload and better image quality [11,12,13,14,15].

For the sake of information security and privacy protection, data are usually encrypted before uploading and transmission. The sender encrypts the plaintext by using the keys and sends the encrypted ciphertext to the receiver. Since the ciphertext is sent, the security of the information is ensured. The receiver can decrypt the ciphertext into plaintext based on the obtained keys. The secret homomorphism was proposed by Rivest in 1978 [16]. First, several plaintexts were encrypted, then the encrypted ciphertexts could be multiplied or added, and decrypted finally. After experimental verification, the results of the operations performed under the idea has been consistent with the results of performing the same operations directly on the same plaintext. There have been more developments in the design of homomorphic encryption schemes. Because of the particularity of the encryption scheme, the homomorphic encryption technology can perform data operations in the encrypted domain without affecting the final decrypted data. Therefore, homomorphic encryption technologies have been widely used in the operation of secure data. The common homomorphic encryption technology had Pallier encryption system [17], RSA encryption system [18] and ElGmal encryption system [19]. Reversible data hiding technique in encrypted domain is based on this property. Reversible data hiding technique in encrypted domain can implement data hiding procedure in encrypted domain and can recover the original plaintext without error after decryption and data extraction. Owing to this merit, reversible data hiding in encrypted images has been a research hotspot in information security community recently.

There are several categories of reversible data hiding techniques in the literature. The first category is difference expansion-based algorithm, originally proposed by Tian [4]. The reversible data hiding algorithms based on the difference expansion algorithm utilize the correlation of the pixel values of adjacent pixels, and replace the original pixels with the difference of adjacent pixels. After that, the other category of reversible data hiding algorithms was proposed by Thodi [20] and then developed by Li et al. [12]. There are several methods for reversible data hiding in encrypted domain. In [21], by using absolute mean difference of multiple neighboring pixels, the authors proposed a reversible data hiding algorithm in encrypted domain. In [22], the authors proposed another algorithm based on discrete fourier transform and compressive sensing in encrypted domain. In [23,24], the authors proposed two methods for reversible data hiding by using Paillier cryptosystem. In [23], two adjacent pixels as a group are encrypted and then the differences of the two adjacent pixels in each group are computed to generate a difference histogram. The information can be hiding into the histogram by using histogram shifting technique and the homomorphic properties of Paillier system.

In recent years, image encryption technology has developed rapidly. Li [25] briefly summarized the design of image encryption schemes and made an analysis of the challenge of the image encryption faced in the future. In this paper, we propose a new reversible data hiding scheme in the fully homomorphic encrypted domain with the DGHV public key cryptosystem which was proposed by Dijk, Gentry, Halevi and Vaikuntanathan in [26]. By analyzing the insufficiency of existing homomorphic encryption systems, the homomorphic encryption scheme has been improved for multiple bits data encryption in [27]. With the improved encryption scheme, in an image, the use of two adjacent pixels as a group is encrypted by using the same random number. After that, the difference of the two adjacent pixels in each group was computed for computation of histogram. In the encrypted domain, additional data can be embedded and extracted by shifting the difference histogram. Without the data hiding key, the embedded data cannot be extracted. In addition, the additional data can be extracted from the directly decrypted image and the original image can be restored. Compared with the the work in [23,24], the proposed scheme has no preprocessing operations and has lower computational cost.

The remainer of this paper is organized as follows. Shannon’s information entropy theory in combination with the encrypted domain is introduced in Section 2. A brief introduction of fully homomorphic encryption over the integers is given in Section 3. The details of the proposed reversible data hiding scheme is introduced in Section 4. Experiment results are given in Section 5. Finally, we have a conclusion in Section 6.

## 2. Shannon Information Theory

In 1948, Shannon put forward the mathematical theory of communication, which initiated the study of modern information theory [28]. He considered that information entropy can be used to measure the probability distribution of the pixels of a grayscale image. The larger is the information entropy, the more uniform is the probability distribution of the gray image pixels. In the Shannon’s information theory, the set of different states of the message samples in the information source is called the probability space, which can be represented by *X*. In the probability space, the probability of occurrence of the samples is different, and their uncertainty is different. The greater is the probability of a sample appearing, the smaller is its uncertainty; conversely, the smaller is the probability of a sample appearing, the greater is its uncertainty. If the probability of sample $x_{i}$ is $p(x_{i})$, the information entropy is defined as

(1) $$H\left(X\right)=-\sum_{i}p\left(x_{i}\right)\log p\left(x_{i}\right)$$

where H(X) is called information entropy. As is known, the encrypted image loses the correlation between pixels and increases the information entropy of the image. The increase of the entropy makes the histogram distribution of image more uniform. Li [29] pointed out that the information entropy of encrypted image tends to the maximum, thus extra information is difficult to be embedded in the encrypted domain.

In the proposed algorithm, we used two adjacent pixels as a group and encrypted the two pixels in a group with the same parameter. In such a way, the correlation between adjacent pixels can be transmitted to the encrypted domain. As a result, the difference histogram distribution of the encrypted image is not uniform. Thus, there is a residual entropy space for additional information embedding in the encrypted domain.

## 3. Fully Homomorphic Encryption over the Integers

DGHV fully homomorphic encryption over the integers, which has the characteristics of additive homomorphism and multiplicative homomorphism, is widely used in the field of security. In this cryptosystem, a plaintext is encrypted with public keys. The plaintext can be retrieved after decrypting corresponding ciphertexts with private keys. To ensure the security of the encryption system, the proposed algorithm introduces the greatest common divisor (GCD) problem. Since additive homomorphism and multiplicative homomorphism are permitted in this cryptosystem, it provides an efficient approach to process the original data in encrypted domain.

### 3.1. Key Generation

Select two integers *p* and *q*. *p* is an odd number and is used as the private key, and *q* is the large integer. For the security of the ciphertexts, the two integers *p* and *q* must satisfy q>>p. The greatest common divisor problem is introduced by adding a number of ciphertexts $x_{i}\left(0\leq i\leq l\right)$ with plaintexts of 0, so that the value of ciphertext is large, to ensure it is not easy to decrypt the ciphertext. At the same time, we must ensure that $x_{0}$ is the largest. The public key is $pk,pk=<x_{0},x_{1},\ldots ,x_{l}>$.

### 3.2. Encryption

For each original data *m*, select an integer *r* randomly. *r* is an integer that is generated randomly during the encryption process and *n* is the number of bits encrypted at one time. Private key *p* must satisfy

(2) $$m+2^{n}r<\frac{p}{2}$$

Denote the encryption function as E[·]. The corresponding ciphertext *c* can be obtained by

(3) $$c=E\left[m\right]=m+2^{n}r+pq$$

The greatest common divisor problem is introduced by adding a number of ciphertexts with a plaintext of 0, so that the value of ciphertext is large, and the ciphertext is not easy to be decrypted. Equation (3) can be formulated as Equation (4) by introducing the greatest common divisor problem

(4) $$c=E\left[m\right]=\left(m+2^{n}r+\sum_{i\in S}x_{i}\right)\;mod\;x_{0}$$

where *c* represents the ciphertext of *m* after adding the greatest common divisors $\sum_{i\in S}x_{i}$, and *S* is a subset of {0,1,…,l}.

### 3.3. Decryption

With corresponding private key *p*, the original plaintext *m* can be derived by

(5) $$m=D\left[c\right]=\left(c\;mod\;p\right)\;mod\;2^{n}$$

where the decryption function is denoted as D[·].

### 3.4. Homomorphic Addition

According to Equations (3) and (5), we have the following derivations for the ciphertexts of $m_{1}$ and $m_{2}$:

(6) $$E\left[m_{1}\right]=\left(m_{1}+2^{n}r_{1}+pq_{1}\right)$$

(7) $$E\left[m_{2}\right]=\left(m_{2}+2^{n}r_{2}+pq_{2}\right)$$

(8) $$E\left[m_{1}\right]+E\left[m_{2}\right]=\left(m_{1}+m_{2}\right)+2^{n}\left(r_{1}+r_{2}\right)+p\left(q_{1}+q_{2}\right)$$

(9) $$\begin{array}{c} D[E\left[m_{1}\right]+E\left[m_{2}\right]]\\ =[\left(\left(m_{1}+m_{2}\right)+2^{n}\left(r_{1}+r_{2}\right)+p\left(q_{1}+q_{2}\right)\right)\;mod\;p]\;mod\;2^{n}\\ =\left(\left(m_{1}+m_{2}\right)+2^{n}\left(r_{1}+r_{2}\right)\right)\;mod\;2^{n}\\ =m_{1}+m_{2} \end{array}$$

where the result of encryption after addition of plaintexts $m_{1}$ and $m_{2}$ is the same as the result of encrypting the plaintexts $m_{1}$ and $m_{2}$ and then adding in the ciphertext domain.

### 3.5. Homomorphic Multiplication

According to Equations (3) and (5), we have the following derivations for the ciphertexts of $m_{1}$ and $m_{2}$:

(10) $$\begin{array}{c} E\left[m_{1}\right]\times E\left[m_{2}\right]=\left(m_{1}+2^{n}r_{1}\right)\left(m_{2}+2^{n}r_{2}\right)+p\left(\left(m_{1}+2^{n}r_{1}\right)q_{2}+\left(m_{2}+2^{n}r_{2}\right)q_{1}+pq_{1}q_{2}\right) \end{array}$$

(11) $$\begin{array}{c} D[E\left[m_{1}\right]\times E\left[m_{2}\right]]\\ =[\left(\left(m_{1}+2^{n}r_{1}\right)\left(m_{2}+2^{n}r_{2}\right)+p\left(\left(m_{1}+2^{n}r_{1}\right)q_{2}+\left(m_{2}+2^{n}r_{2}\right)q_{1}+pq_{1}q_{2}\right)\right)\;mod\;p]\;mod\;2^{n}\\ =\left(\left(m_{1}+2^{n}r_{1}\right)\left(m_{2}+2^{n}r_{2}\right)\right)\;mod\;2^{n}\\ =\left(m_{1}m_{2}+2^{n}\left(m_{1}r_{2}+m_{2}r_{1}+2^{n}r_{1}r_{2}\right)\right)\;mod\;2^{n}\\ =m_{1}m_{2} \end{array}$$

where the result of encryption after multiplication of plaintexts $m_{1}$ and $m_{2}$ is the same as that of multiplication of ciphertext after encryption of plaintext $m_{1}$ and $m_{2}$. In conclusion, DGHV homomorphic encryption satisfies both additive homomorphism and multiplicative homomorphism, which means that DGHV is a fully homomorphic encryption scheme.

## 4. Reversible Data Hiding Scheme with Public Key Cryptosystem

**Figure 1:**
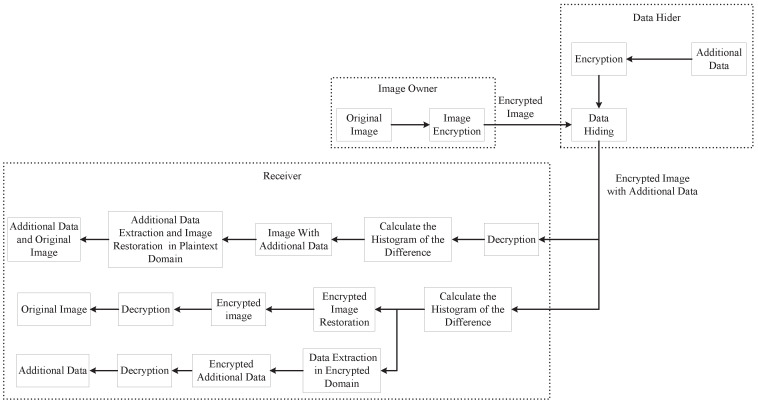
Sketch of the proposed reversible data hiding scheme with public key cryptography.

Figure 1 plots the sketch of the proposed scheme, which is composed of four main phases: image encryption, data hiding, data extraction and image restoration. For the data hiding algorithm, refer to [23]. Reversible data hiding is an effective authentication or content integrity verification technique in which hidden data can be completely extracted and the original carrier can be recovered non-destructively after data extraction [30]. After the image owner encrypts the image, the data hider will embed the hidden data in the encrypted domain. With the private key, the receiver can use the corresponding decryption method to extract the encrypted embedded data. Then, by using the steps of extracting algorithm, the embedded data in the encrypted domain is extracted. Only when the private key is possessed, the receiver can obtain an image containing the embedded data similar to the original image. After decrypting, the embedded data can be extracted and the original image can be restored.

### 4.1. Image Encryption

According to the property of DGHV homomorphic cryptosystem, the parameter r(k) is selected randomly to ensure security. It is difficult to embed additional data directly into an encrypted image, because magnitude relationships among plaintexts cannot be kept to the corresponding ciphertexts. For this reason, we designed a corresponding data hiding strategy to embed additional data in the encrypted domain. With this strategy, we can shift the difference histogram for hiding data in encrypted domain.

Firstly, groups of two selected pixels are chosen from original image. Denote the two pixels in *k*th group as $P_{1}\left(k\right)$ and $P_{2}\left(k\right)$. A data owner selects an integer r(k) randomly, and encrypts $P_{1}\left(k\right)$ and $P_{2}\left(k\right)$ with the public key $\sum_{i\in S(k)}x_{i}$,

(12) $$C_{1}\left(k\right)=E\left(m_{1}\right)=\left(m_{1}+2^{n}r_{k}+\sum_{i\in S(k)}x_{i}\right)\;mod\;x_{0}$$

(13) $$C_{2}\left(k\right)=E\left(m_{2}\right)=\left(m_{2}+2^{n}r_{k}+\sum_{i\in S(k)}x_{i}\right)\;mod\;x_{0}$$

where $C_{1}\left(k\right)$ and $C_{2}\left(k\right)$ are the corresponding ciphertexts of $P_{1}\left(k\right)$ and $P_{2}\left(k\right)$, respectively, and S(k) is a subset of {0,1,…,l} in the *k*th group. We let n=9 in this paper to ensure correct decryption. To ensure the security of the ciphertext, we select an integer $r_{2}$ in each group, which satisfies $r_{2}=i$ and encrypt 0 to generate $C_{2}{\left(k\right)}^{\prime }$,

(14) $$C_{2}{\left(k\right)}^{\prime }=C_{2}\left(k\right)+E\left[0,r_{2}\left(k\right)\right]=C_{2}\left(k\right)+C\left(0\right)$$

Let D[C(k)] be the decrypted version of C(k) and D[C(k)] satisfies

(15) $$D\left[C\left(k\right)\right]=\left(C\left(k\right)\;mod\;p\right)\;mod\;2^{n}$$

A data owner encrypts the original image, and a data hider can obtain the encrypted image and $r_{2}$. The embedded data are also encrypted in this method.

### 4.2. Data Hiding

With the same method of the data encryption, the additional data can be encrypted. Data hider calculates the difference of the two pixels of a group, and then calculates the positive peak point and the negative peak point of the histogram. The pixels corresponding to the two peak points are used to embed additional data.

### 4.3. Data Embedding

After the image owner encrypts the original image by using the public key pk, the encrypted image $I_{m}$ and $r_{2}$ is transmitted to the data hider. When the data hider receives the encrypted image, the data hider obtains C(0) by receiving $r_{2}$, and makes $C_{2}{\left(k\right)}^{\prime }$ subtract C(0) to recover $C_{2}\left(k\right)$. The $C_{2}\left(k\right)$ can be calculated by

(16) $$C_{2}\left(k\right)=C_{2}{\left(k\right)}^{\prime }-C\left(0\right)$$

Then, the data hider embeds the additional data in the encrypted image and obtains a new image $I_{w}$. Finally, the data hider sends $I_{w}$ and $r_{2}$, the position of the positive peak point of the histogram and the position of the negative peak point of the histogram to the receiver, and the receiver can extract the embedded data and restore the original image with private keys.

In the proposed algorithm, the adjacent two encrypted pixels $C_{1}\left(k\right)$ and $C_{2}\left(k\right)$ are subtracted to obtain $C_{d}\left(k\right)$. Then, the position of the positive peak point of the histogram is recorded as $EC_{max}$, and the position of the negative peak point of the histogram is recorded as $EC_{min}$. When $C_{d}\left(k\right)=EC_{max}$, one bit of encrypted additional data is embedded in the adjacent encrypted pixel $C_{1}\left(k\right)$, and, when $C_{d}\left(k\right)=EC_{min}$, one bit of encrypted additional data is embedded in the adjacent encrypted pixel $C_{2}\left(k\right)$. In this paper, only one round of extra information is embedded.

The specific additional data embedding steps are shown as follows. Firstly, the difference between adjacent pixels in the encrypted domain is calculated as

(17) $$C_{d}\left(k\right)=C_{1}\left(k\right)-C_{2}\left(k\right).$$

**Figure 2:**
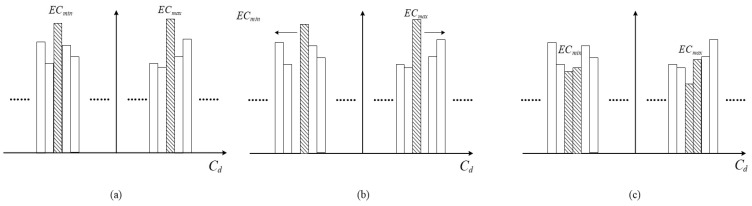
Embedding additional information by shifting the histogram: (**a**) histogram of the differences before embedding; (**b**) shifting the difference histogram to free up embedding space; and (**c**) histogram after embedding the additional information.

Secondly, the position of the positive peak point of the histogram and the position of the negative peak point of the histogram are selected, respectively. When $C_{1}\left(k\right)⩾C_{2}\left(k\right)$, the additional data embeds in the right half of the histogram. When $C_{1}\left(k\right)<C_{2}\left(k\right)$, the additional data embeds in the left half of the histogram. The histogram shifting process is shown in Figure 2.

Thirdly, the additional information is embedded in the carrier image in the encrypted domain by shifting the difference histogram. When the additional data is encrypted, we denote it as $E_{w}\left(w\right)$. In this paper, the same public key $\sum_{i\in S(k)}x_{i}$ and random number r(k) are used to encrypt the additional information. Bit 1 and bit 0 are denoted as E(0) and E(1), respectively, in the encrypted domain and encrypted by the public key, which is the same as the public key used to encrypt the additional information. The embedded image pixels are $C_{w1}\left(k\right)$ and $C_{w2}\left(k\right)$. $C_{w1}\left(k\right)$ and $C_{w2}\left(k\right)$ are calculated as follows:

If $C_{1}\left(k\right)⩾C_{2}\left(k\right)$,

(18) $$C_{w1}\left(k\right)=\left\{\begin{array}{cc} \left(C_{1}\left(k\right)+E_{w}\left(w\right)\right)\;mod\;x_{0}, & if\;C_{d}\left(k\right)=EC_{max}\\ \left(C_{1}\left(k\right)+E\left(1\right)\right)\;mod\;x_{0}, & if\;C_{d}\left(k\right)⩾EC_{max}+1\\ \left(C_{1}\left(k\right)+E\left(0\right)\right)\;mod\;x_{0}, & else \end{array}\right.$$

(19) $$C_{w2}\left(k\right)=\left(C_{2}\left(k\right)+E\left(0\right)\right)\;mod\;x_{0}$$

else

(20) $$C_{w2}\left(k\right)=\left\{\begin{array}{cc} \left(C_{2}\left(k\right)+E_{w}\left(w\right)\right)\;mod\;x_{0}, & if\;C_{d}\left(k\right)=EC_{min}\\ \left(C_{2}\left(k\right)+E\left(1\right)\right)\;mod\;x_{0}, & if\;C_{d}\left(k\right)⩽EC_{min}-1\\ \left(C_{2}\left(k\right)+E\left(0\right)\right)\;mod\;x_{0}, & else \end{array}\right.$$

(21) $$C_{w1}\left(k\right)=\left(C_{1}\left(k\right)+E\left(0\right)\right)\;mod\;x_{0}$$

Denote $P_{w1}\left(k\right)$ and $P_{w2}\left(k\right)$ as the plaintext versions of $C_{w1}\left(k\right)$ and $C_{w2}\left(k\right)$, respectively. The effect of data hiding on plaintexts is to change $P_{1}\left(k\right)$ and $P_{2}\left(k\right)$ to $P_{w1}\left(k\right)$ and $P_{w2}\left(k\right)$:

If $P_{1}\left(k\right)⩾P_{2}\left(k\right)$,

(22) $$P_{w1}\left(k\right)=\left\{\begin{array}{cc} P_{1}\left(k\right)+w, & if\;C_{d}\left(k\right)=EC_{max}\\ P_{1}\left(k\right)+1, & if\;C_{d}\left(k\right)⩾EC_{max}+1\\ P_{1}\left(k\right), & else \end{array}\right.$$

(23) $$P_{w2}\left(k\right)=P_{2}\left(k\right)$$

else

(24) $$P_{w2}\left(k\right)=\left\{\begin{array}{cc} P_{2}\left(k\right)+w, & if\;C_{d}\left(k\right)=EC_{min}\\ P_{2}\left(k\right)+1, & if\;C_{d}\left(k\right)⩽EC_{min}-1\\ P_{2}\left(k\right), & else \end{array}\right.$$

(25) $$P_{w1}\left(k\right)=P_{1}\left(k\right)$$

To ensure the security of the ciphertext, according to Equations (14), we generate $C_{w2}{\left(k\right)}^{\prime }$ with $r_{2}$ in each group,

(26) $$C_{w2}{\left(k\right)}^{\prime }=C_{w2}\left(k\right)+E\left[0,r_{2}\left(k\right)\right]=C_{w2}\left(k\right)+C\left(0\right)$$

It can be seen that the embedding algorithm can be used not only in the plaintext domain, but also in the ciphertext domain. In other words, the data owner sends the encrypted image to the data hider, and then the data hider embeds the encrypted additional data into the encrypted image directly.

### 4.4. Data Extraction and Image Restoration

In the proposed scheme, data extraction and image restoration can be completed together. There are two ways to extract the hidden data and restore the original image.

#### 4.4.1. Extract the Hidden Data and Restore Original Ciphertext Image in Encrypted Domain

When the receiver receives the encrypted embedded image, $r_{2}$, the position of the positive and the negative peak point of the histogram, which has 18 bits of side information, the receiver can find the embedded position and use the following method to extract the embedded additional data and restore original pixel:

Firstly, the receiver obtains C(0) by receiving $r_{2}$, and makes $C_{w2}{\left(k\right)}^{\prime }$ subtract C(0) to recover $C_{w2}\left(k\right)$. The $C_{w2}\left(k\right)$ can be calculated by

(27) $$C_{w2}\left(k\right)=C_{w2}{\left(k\right)}^{\prime }-C\left(0\right)$$

Secondly, calculate the difference between adjacent pixels:

(28) $$C_{wd}\left(k\right)=C_{w1}\left(k\right)-C_{w2}\left(k\right)$$

where $C_{wd}\left(k\right)$ is denoted as the difference between adjacent pixels, which contain the additional data in the encrypted domain.

Thirdly, extract embedded data and restore original pixels:

(29) $$E_{w}\left(w\right)=\left\{\begin{array}{cc} E_{w}\left(0\right), & if\;C_{wd}\left(k\right)=EC_{max}\;or\;C_{wd}\left(k\right)=EC_{min}\\ E_{w}\left(1\right), & if\;C_{wd}\left(k\right)=EC_{max}+1\;or\;C_{wd}\left(k\right)=EC_{min}-1 \end{array}\right.$$

If $C_{w1}\left(k\right)\geq C_{w2}\left(k\right)$,

(30) $$C_{1}\left(k\right)=\left\{\begin{array}{cc} \left(C_{w1}\left(k\right)-E\left(1\right)\right)\;mod\;x_{0}, & if\;C_{wd}\left(k\right)⩾EC_{max}+1\\ \left(C_{w1}\left(k\right)-E\left(0\right)\right)\;mod\;x_{0}, & else \end{array}\right.$$

(31) $$C_{2}\left(k\right)=\left(C_{w2}\left(k\right)-E\left(0\right)\right)\;mod\;x_{0}$$

else

(32) $$C_{2}\left(k\right)=\left\{\begin{array}{cc} \left(C_{w2}\left(k\right)-E\left(1\right)\right)\;mod\;x_{0}, & if\;C_{wd}\left(k\right)⩽EC_{max}-1\\ \left(C_{w2}\left(k\right)-E\left(0\right)\right)\;mod\;x_{0}, & else \end{array}\right.$$

(33) $$C_{1}\left(k\right)=\left(C_{w1}\left(k\right)-E\left(0\right)\right)\;mod\;x_{0}$$

(34) $$W\left(w\right)=\left(E_{w}\left(w\right)\;mod\;p\right)\;mod\;2^{n}$$

(35) $$P\left(k\right)=\left(C_{I}\left(k\right)\;mod\;p\right)\;mod\;2^{n}$$

where W(w) represents the extracted additional data after decryption, *p* is the private key and P(k) is the restored image after decryption. It can be seen that the receiver can extract the additional data and restore original image without any losses.

#### 4.4.2. Extract the Hidden Data and Restore the Original Image after Decryption

When the receiver receives the encrypted embedded image, the position of the positive peak point of the histogram and the position of the negative peak point of the histogram, the receiver can find the embedding position and use the following method to extract the embedded additional data and restore the original image:

Firstly, decrypt the encrypted embedded image by:

(36) $$P_{w}\left(k\right)=\left(C_{w}\left(k\right)\;mod\;p\right)\;mod\;2^{n}$$

where $P_{w}\left(k\right)$ represents the decrypted image including the additional data in the plaintext domain.

Secondly, calculate the differences between adjacent pixels:

(37) $$P_{wd}\left(k\right)=P_{w1}\left(k\right)-P_{w2}\left(k\right)$$

where $P_{wd}\left(k\right)$ is denoted as the difference between the adjacent pixels in the plaintext domain.

Finally, extract embedded data with:

(38) $$W\left(k\right)=\left\{\begin{array}{cc} 1, & if\;P_{wd}\left(k\right)=EP_{max}+1\;or\;P_{wd}\left(k\right)=EP_{min}-1\\ 0, & if\;P_{wd}\left(k\right)=EP_{max}\;or\;P_{wd}\left(k\right)=EP_{min} \end{array}\right.$$

If $P_{w1}\left(k\right)\geq P_{w2}\left(k\right)$.

(39) $$P_{1}\left(k\right)=\left\{\begin{array}{cc} P_{w1}\left(k\right)-1, & if\;P_{wd}\left(k\right)⩾EP_{max}+1\\ P_{w1}\left(k\right), & else \end{array}\right.$$

(40) $$P_{2}\left(k\right)=P_{w2}\left(k\right)$$

else

(41) $$P_{2}\left(k\right)=\left\{\begin{array}{cc} P_{w2}\left(k\right)-1, & if\;P_{wd}\left(k\right)⩽EP_{min}-1\\ P_{w2}\left(k\right), & else \end{array}\right.$$

(42) $$P_{1}\left(k\right)=P_{w1}\left(k\right)$$

However, since the value range of the grayscale image pixel in the plaintext domain is [0,255], the pixel value of some images restored to the plaintext domain may be 256 after decryption, which is the overflowing problem. At this point, we change the pixel value 256 to 255, and restore their position information. Then, we can embed these positions into the watermarked image through a reversible algorithm. On the receiver side, the receiver can recover the image by extracting the position information with the reversible algorithm. After that, the legal receiver can extract the additional data and restore the original image without any loss.

## 5. Experimental Results

In the experiment, we objectively evaluated the experimental results from the aspects of computational cost, peak signal-to-noise ratio (PSNR), embedded rate and imperceptibility. At the same time, by comparing with Xiang [23] and Xiang [24], it showed that the DGHV fully homomorphic encryption watermarking algorithm proposed in this paper is greatly improved in terms of computational cost.

### 5.1. Computational Cost

A series of experiments confirmed the performance of proposal algorithm. Different standard test images were used to verify the results of the experiment. One of the important performance indicators for measuring the homomorphic encryption algorithms is computational cost. The lower is the computational cost, the more useful the program is in the application. Table 1 presents four grayscale images of size 512 × 512 for testing and shows the average computational cost of ten times under different capacity.

On the one hand, it can be seen from the experimental data in Table 1 that the encryption and decryption times of different images are slightly different while the embedding capacity and the carrier image size are the same. Besides, the embedding and extraction times are also slightly different, respectively, which are mainly due to the different images in the text. On the other hand, at the stage of embedding additional data and extracting data, the computational cost increases as the number of embedded data increases.

By comparing a plaintext and the resulting version from the corresponding ciphertext, the results show that the pixel values of the original image are the same as the pixel values of the image pixels after decryption. As for the extraction of the additional data, there is no data loss or disorder of the sequence in the extraction of the embedded data.

Table 2 uses three same images, respectively, and selects Lena diagrams but different size as a group to compare the average computational cost of ten times of embedded additional data with different number of bits. In Table 2, it can be seen that the computational cost of the proposed algorithm corresponds to the size of the embedded data and the original image. For the same embedding rate, the smaller is the carrier image size, the lower is the computational cost. Furthermore, the computational cost is positively related to the size of the original image. The larger is the image, the higher is the computational cost needed for the encryption. The size of the embedded data has less influence on the computational cost in comparison with the image size.

### 5.2. Security

The security of hiding information in the encrypted domain is one of the important issues. The algorithm proposed in this paper is an algorithm for hiding information in the DGHV full homomorphic encrypted domain, which has both the property of addition homomorphism and multiplication homomorphism. To ensure the security of additional information, the greatest common divisor problem is introduced in the proposed algorithm. That is to say, some ciphertext sets $x_{i}$ with zero in the plaintext are added. Let $x_{i}$ be the public key set and randomly select a subset of $x_{i}$ as the public key for the encryption. Because the additive subsets are ciphertext with 0 in the plaintext, these subsets have no effect on the decryption. If an attacker does not know the private key, he cannot decrypt the encrypted image and cannot extract the additional information since the plaintexts were encrypted with the random integer r(k). Therefore, the security of the embedded information is ensured.

### 5.3. Imperceptibility

PSNR is one of the important indicators to measure the imperceptibility of the watermarking algorithm in the spatial domain. Generally speaking, the larger is the PSNR, the better is the imperceptibility achieved. Table 3 lists the PSNR values with four different images sized by 512 × 512. We can see that all of the PSNR values are over 50 dB, showing the embedding distortion is smaller. In Table 3, it can be seen that, when the embedding capacity is larger, the PSNR decreases accordingly.

### 5.4. Original Images and Their Processed Versions

**Figure 3:**
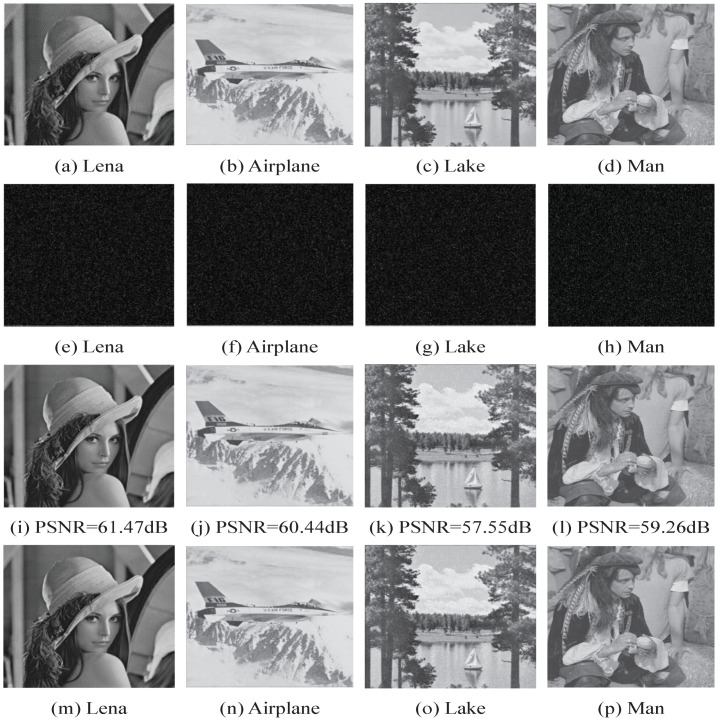
Four original test images Lena, Airplane, Lake and Man (**a**–**d**); the four encrypted images with embedded additional data (**e**–**h**); the four decrypted images (**i**–**l**); and the four restored images (**m**–**p**).

Figure 3 plots the four original images (Lena, Airplane, Lake and Man), the encrypted images after watermarking, the directly decrypted images, and the restored images. In total, 4096 bits of data are embedded in the encrypted images, respectively. We have the following observations from this figure:

(1) The encrypted image after embedding data has no correlation with the original image. The experimental results show that the additional information in the DGHV fully homomorphic encrypted domain has achieved good results, which depends on the security of DGHV fully homomorphic encryption. That is, without the private key, the encrypted image cannot be decrypted and the additional information cannot be extracted in the encrypted domain.
(2) Comparing the original image with the decrypted image containing the additional information, the visual distortion due to the watermark is small. After the original images are encrypted and embedded with 4096 bits of data, the PSNR values of the watermarked images are greater than 57 dB in Figure 3, which has satisfactory imperceptibility.
(3) The information embedded in the fully homomorphic encrypted domain in the proposed algorithm is reversible, and the original image can be successfully recovered without distortion after decrypting and extracting the embedded information.

### 5.5. Performance Comparison

The proposed algorithm in this paper uses the histogram shifting technique to embed additional information in the DGHV encryption domain. Compared with Xiang [23], the algorithm proposed in this paper has lower computational cost.

**Figure 4:**
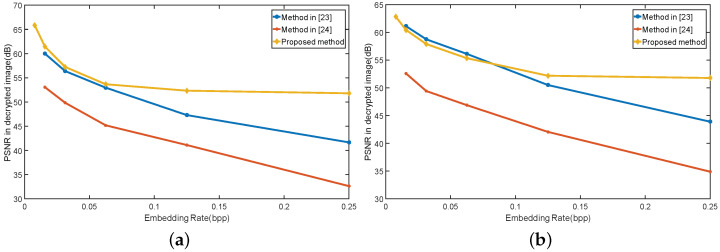
Comparison of embedding capacity versus embedding distortion in different images: (**a**) lena; and (**b**) airplane.

To compare the proposed method with two existing methods [23,24], Figure 4 plots the PSNR values at different embedding rates by using the Lena image and the Airplane image, respectively. It can be seen in Figure 4 that, at the low embedding rate, the PSNR is slightly different from that of the method in [23]. At the high embedding rate, the PSNR of the proposed algorithm is higher than that of the method in [23]. In addition, the PSNR value of the proposed method is much higher than that of the method in [24]. Compared with Xiang [24], the proposed algorithm has greatly improved the imperceptibility of the image after embedding for the same embedding rate.

## 6. Conclusions

In this paper, we propose a reversible data hiding algorithm with DGHV fully homomorphic encryption and analyzed the feasibility of the scheme from the perspective of information entropy. In the proposed algorithm, we used two adjacent pixels as a group and encrypted the two pixels in a group with the same parameter. In such a way, the correlation between adjacent pixels can be transmitted to the encrypted domain. As a result, the difference histogram distribution of the encrypted image is not uniform. Thus, there is a residual entropy space for additional information embedding in the encrypted domain.

This method has better solved the problems of quickly encrypting multiple bits of data and embedding additional data in an encrypted domain. This algorithm has lower computational cost, higher security, and better imperceptibility. In future research, how to embed a robust and reversible watermark into this encrypted domain will be considered.

## Figures and Tables

**Table 1:** Computational cost for different embedded bits.

Picture	Embedded Data Bits			Time (s)			
		Encryption	Embed	Extraction (Encrypted Domain)	Extraction (Plaintext Domain)	Decryption (Embedded Image)	Decryption (Original Image)
Lena	1024	1.5094	0.0309	0.0270	0.0264	0.0019	0.0019
Airplane		1.5095	0.0460	0.0388	0.0395	0.0021	0.0020
Lake		1.5102	0.0737	0.0638	0.0639	0.0019	0.0020
Man		1.5065	0.0455	0.0387	0.0398	0.0019	0.0019
Lena	2048	1.5103	0.0443	0.0395	0.0399	0.0021	0.0020
Airplane		1.5078	0.0584	0.0505	0.0520	0.0019	0.0019
Lake		1.4905	0.0875	0.0724	0.0753	0.0019	0.0019
Man		1.5257	0.0636	0.0552	0.0566	0.0018	0.0018
Lena	4096	1.5106	0.0707	0.0648	0.0667	0.0018	0.0019
Airplane		1.5125	0.0795	0.0730	0.0740	0.0019	0.0020
Lake		1.5231	0.1244	0.1078	0.1128	0.0019	0.0019
Man		1.5261	0.0960	0.0879	0.0907	0.0021	0.0019

**Table 2:** Computational cost with the Lena image with different sizes and embedded rates.

Size	Embedded Data Bits			Time (s)			
		Encryption	Embed	Extraction (Encrypted Domain)	Extraction (Plaintext Domain)	Decryption (Embedded Image)	Decryption (Original Image)
256 × 256	1024	0.3784	0.0125	0.0106	0.0107	0.0016	0.0017
512 × 512		1.5094	0.0309	0.0270	0.0264	0.0019	0.0019
1024 × 1024		6.0922	0.4456	0.4242	0.4194	0.0069	0.0071
256 × 256	2048	0.3745	0.0230	0.0210	0.0213	0.0016	0.0016
512 × 512		1.5103	0.0443	0.0395	0.0399	0.0021	0.0020
1024 × 1024		6.0482	0.4462	0.4262	0.4243	0.0066	0.0076
256 × 256	4096	0.3772	0.0473	0.0456	0.0469	0.0016	0.0016
512 × 512		1.5106	0.0707	0.0648	0.0667	0.0018	0.0019
1024 × 1024		6.0841	0.4533	0.3805	0.4347	0.0067	0.0068

**Table 3:** PSNR values with the different embedding capacity.

Picture	Embedded Data Bits	PSNR (dB)	Picture	Embedded Data Bits	PSNR (dB)
Lena	2048	65.8630	Lake	2048	59.5992
	4096	61.4712		4096	57.5459
	8192	57.2428		8192	54.9346
	16384	53.6700			
Airplane	2048	62.8160	Man	2048	62.1950
	4096	60.4387		4096	59.2608
	8192	57.9015		8192	56.2677
	16384	55.3511		16384	53.6685

## Abbreviations

The following abbreviations are used in this manuscript:

DGHV	Dijk, Gentry, Halevi and Vaikuntanathan
GCD	Greatest Common Divisor
PSNR	Peak Signal-to-noise Ratio
